# Phosphorylation of SNX17 impedes activation of Retriever-mediated sorting

**DOI:** 10.1016/j.jbc.2025.110222

**Published:** 2025-05-09

**Authors:** Jan Dominik Speidel, Kaikai Yu, Ralph Thomas Böttcher

**Affiliations:** Department of Molecular Medicine, Max Planck Institute of Biochemistry, Martinsried, Germany

**Keywords:** sorting nexin, integrin, phosphorylation, phox domain, Retriever, endosome, intracellular trafficking

## Abstract

Sorting nexin 17 (SNX17) functions as cargo receptor on endosomal membranes that enables the recycling of numerous membrane cargo proteins by binding to the Retriever complex. Yet, little is known how SNX17 activity or its membrane recruitment is regulated. Here, we report that phosphorylation of SNX17 at serine 38 (Ser38) within the phox domain serves as a critical regulatory switch governing its endosomal localization and function. A mutant form mimicking the phosphorylated state disrupts SNX17’s ability to bind phosphatidylinositol-3-phosphate, which in turn impairs its association with early endosomal membranes and inactivates SNX17-dependent cargo-recycling in cells. Furthermore, our results demonstrate that Ser38 is part of an autoinhibitory mechanism to regulate SNX17 cargo binding. Collectively, these findings provide new insights into the dynamic regulation of SNX17 activity and Retriever-mediated sorting processes. It also highlights SNX17 Ser38 phosphorylation as a critical regulatory mechanism that controls SNX17’s endosomal localization and cargo recycling function.

Intracellular protein trafficking is a fundamental process essential for cellular homeostasis and function. This complex system is governed by the endosomal network, a series of membrane-bound compartments which sorts and transports integral membrane proteins and their associated molecules. This intricate machinery involves multiple protein complexes that coordinate the recognition, sorting, and transport of cargo proteins throughout the cell (1, 2, 3). To date, two major recycling pathways from endosomes to the plasma membrane have been discovered. One relies on sorting nexin 27 (SNX27) and the well-studied Retromer complex (4, 5), the other is based on sorting nexin 17 (SNX17) and the Retriever complex (6).

The Retriever complex plays a critical role in the recycling of a broad spectrum of plasma membrane proteins, including tyrosine receptor kinases, integrins, G-protein coupled receptors, and lipoprotein receptors (6, 7, 8). The Retriever is a trimeric complex that consists of VPS35L, VPS26C, and VPS29 and is structurally related to the Retromer complex (6) but is functionally distinct as it associates with the COMMD–CCDC22–CCDC93 complex (8) to form a larger-order complex known as the Commander complex (8, 9). The Commander complex is essential for efficient cargo sorting and has been implicated in several metabolic disorders (7, 10), viral infections (11, 12, 13), and developmental syndromes (14, 15). Cargo recognition and tethering to the Retriever complex is achieved by the cargo adaptor SNX17 (16, 17).

SNX17 is a member of the sorting nexin (SNX) family of proteins that regulate intracellular trafficking through the endolysosomal system and are characterized by the presence of the lipid-binding phox-homology (PX) domain. Besides the PX domain, SNX17 consists of a 4.1/ezrin/radixin/moesin (FERM) domain and an unstructured carboxy (C)-terminal tail. The PX domain recruits SNX17 to endosomal membranes by binding to phosphatidylinositol 3-phosphate (PI3P), while the FERM domain is crucial for binding cargo proteins containing NxxY/NxxF motifs (18, 19, 20, 21). The C-terminal tail of SNX17 binds directly to a pocket near the VPS35L:VPS26C interface (16, 17) and exhibits an autoinhibitory mechanism that regulates the interaction of the FERM domain with cargo proteins (16). Even before the discovery of the Retriever complex, SNX17 and its paralog SNX31 were implicated in the recycling of a large number of receptors and integral membrane proteins from endosomes to the plasma membrane including the LDLR family (LRP1, VLDLR, ApoER2) (22, 23, 24, 25), the amyloid precursor protein (26), and P-selectin (27, 28). Integrins, particularly those of the β1 family, represent one of the best-characterized cargo proteins of SNX17 (6, 29, 30, 31). Integrins are extracellular matrix transmembrane receptors that exist as heterodimers composed of α- and β-subunits. On early endosomes, both SNX17 and SNX31 interact with the β1-integrin intracellular domain and prevent integrin degradation by recycling them back to the cell surface. This recycling mechanism maintains optimal β1-integrin surface levels and enhances integrin-dependent cellular processes, including adhesion and migration (6, 29, 30, 31). Despite SNX17’s role in regulating the endosomal sorting of numerous proteins (19, 31) and retrieving autophagosomal membrane components as part of the recycler complex (32), little is known how SNX17 cargo, membrane, or Retriever binding is regulated. Schaffer *et al*. showed that the energy-sensitive AMP-activated kinase (AMPK) directly phosphorylates serine 437 (Ser437) in the C-terminal tail of SNX17, which correlates with decreased SNX17 protein levels upon AMPK activation (33). However, the functional impact of this phosphorylation on SNX17 cargo recycling and Retriever binding has not yet been investigated nor are other signals known that dynamically regulate SNX17 activity.

In this study, we investigated the regulatory mechanisms controlling SNX17 function, with a particular focus on how phosphorylation affects its endosomal localization and cargo recycling activities. We demonstrate that phosphorylation of serine 38 (S38) in the PX domain of SNX17 serves as a regulatory switch that, when phosphorylated, inhibits SNX17 membrane association, cargo binding, and as a result SNX17’s ability to recycle cargo proteins. Thus, our findings reveal a novel mechanism for fine-tuning SNX17 activity, which maintains endosomal trafficking homeostasis.

## Results

### Expression levels of SNX17 affect its subcellular distribution and phosphorylation

**Figure 1:**
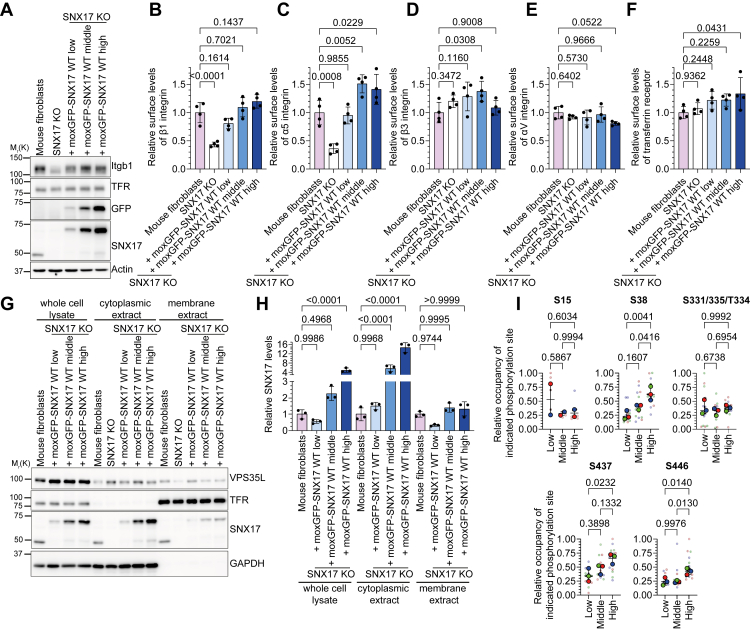
**Expression levels of SNX17 affect its subcellular distribution and phosphorylation status**. *A*, Western blot analysis of WT and SNX17 KO fibroblasts, and SNX17 KO fibroblasts stably re-expressing different levels of moxGFP-tagged WT SNX17 (moxGFP-SNX17 WT). Actin served as loading control. *B*–*F*, quantification of surface levels of Itgb1 (*B*), Itga5 (*C*), Itgb3 (*D*), ItgaV (*E*), and TFR (*F*) on fibroblast cell lines re-expressing different levels of moxGFP-SNX17 WT analyzed by flow cytometry (mean ± s.d.; n = 4). Statistical analysis was carried out with one-way ANOVA + Dunnett multiple comparison test compared to surface levels of mouse fibroblasts. *G*, Western blot analysis of mouse fibroblasts, SNX17 KO cells, and cell lines re-expressing different levels of moxGFP-SNX17 WT before (whole cell lysate) and after fractionation into cytoplasmic and membrane extracts. GAPDH served as a marker protein for the cytoplasmic extract; TFR served as a marker for the membrane extract. *H*, quantification of moxGFP-SNX17 levels in the indicated cell fractions normalized to GAPDH (whole cell lysate and cytoplasmic extract) or to TFR (membrane extract) (mean ± s.d.; n = 3). Statistical analysis was carried out with one-way ANOVA + Šidak multiple comparison test compared to SNX17 levels in mouse fibroblasts of each extract. *I*, relative occupancy of the indicated SNX17 phosphosites in SNX17 KO HeLa cells re-expressing different levels of moxGFP-SNX17 WT (median ± range; n = 3 with five technical replicates each). Statistical analysis was carried out with one-way ANOVA + Tukey multiple comparison test compared to the other samples.

To investigate how SNX17 activity is regulated, we first generated SNX17-deficient cell lines in both mouse fibroblasts and HeLa cells through CRISPR-Cas9–mediated gene deletion. Subsequently, we stably introduced wild-type (WT) moxGFP-tagged SNX17 into these cells using viral vectors and employed fluorescence-activated cell sorting (FACS) to obtain cell populations with low, medium, and high expression levels of the fluorescent fusion protein (Fig. 1*A*, Fig. S1, *A* and *B*). Consistent with previous findings, SNX17 KO reduced α5β1-integrin (Itgb1, Itga5) surface levels which were rescued by re-expression of moxGFP-SNX17 (Fig. 1, *A*C, Fig. S1*B*). Notably, increasing SNX17 expression had only minor effects on α5β1-integrin surface levels (Fig. 1, *B* and *C*) and had no effect on the surface levels of the SNX17-independent cargo proteins Itgb3, ItgaV, and TFR (Fig. 1, *D*–*F*).

These results suggest that increased expression of SNX17 regulates its activity or subcellular localization to mitigate potential disruptions in endosomal cargo trafficking. To investigate this mechanism, we performed cell fractionation to analyze SNX17 distribution between cytoplasmic and membrane compartments in cells expressing low, medium, and high levels of moxGFP-SNX17. While cytoplasmic SNX17 levels increased proportionally with total SNX17 expression, membrane-associated SNX17 remained relatively constant across all cell lines (Fig. 1, *G* and *H*), indicating the presence of mechanisms that prevent excessive membrane recruitment of SNX17 when there are increased levels of expression. The distribution of other endosomal proteins, such as VPS35L, did not change (Fig. 1*G*).

Given the importance of posttranslational modifications, particularly phosphorylation, in protein regulation and cellular signaling, we investigated whether changes in SNX17 phosphorylation might restrict its membrane recruitment when it is highly expressed. We expressed varying amounts of mouse moxGFP-SNX17 in HeLa SNX17 KO cells (Fig. S1*B*) and quantified SNX17 phosphorylation using liquid chromatography-tandem mass spectrometry. Label-free quantification (LFQ) confirmed the differential expression of SNX17 across cell populations (Fig. S1*C*). We consistently detected SNX17 phosphorylation at specific sites across all tested cell lines. These include S38, S437, S446, and either S331, T334, or S335 (Fig. S1, *E-H*). Additionally, in two-thirds of the experiments, we also observed S15 phosphorylation. Interestingly, while the absolute phosphorylation intensity correlated with SNX17 expression levels as expected (Fig. S1, *D*–*H*), the relative occupancy showed site-specific patterns. The proportion of phosphorylated to nonphosphorylated peptides for S38 and S437 increased proportionally with SNX17 expression, whereas it remained stable for S15 and S331/335/T334 (Fig. 1*I*). S446 phosphorylation occupancy increased only at high expression levels, while S409 was detected almost exclusively in its phosphorylated state (data not shown), preventing accurate occupancy calculations. The dose-dependent increase in S38 and S437 phosphorylation occupancy suggested that phosphorylation is a regulatory mechanism controlling SNX17 function. Taken together, these data indicate that SNX17 membrane localization is tightly regulated even at increased levels and point to cellular mechanisms that maintain endosomal trafficking homeostasis.

### SNX17 S38 phosphorylation inhibits cargo protein recycling

The distinct SNX17 phosphorylation patterns at S38 and S437 suggest potential regulatory mechanisms that fine-tune SNX17 function. Since AMPK directly phosphorylates SNX17 at S437 and increases SNX17 turnover (33), we first investigated whether AMPK activation affects SNX17-mediated cargo recycling. We activated AMPK with A-769662 and monitored SNX17 levels over time by Western blotting using phosphorylated acetyl-CoA carboxylase as a marker for AMPK activation (Fig. S2, *A* and *B*). As expected, AMPK activation led to a weak reduction in SNX17 levels after >6 h while the subcellular localization of SNX17 was not affected (Fig. S2, *A*–*C*). To determine whether AMPK-induced SNX17 S437 phosphorylation impacts the stability of SNX17 cargo proteins and potentially impairs cargo recycling, we measured surface β1-integrin stability in control and A-769662-treated fibroblasts using biotin labeling and capture ELISA. We found increased stability of β1 integrin after AMPK activation with different concentrations of A-769662 (Fig. S2*D*), but this increase was SNX17-independent (Fig. S2*E*). This observation likely reflects AMPK's broad regulatory role, as it phosphorylates numerous proteins involved in cell motility, adhesion, and vesicle trafficking (33).

**Figure 2:**
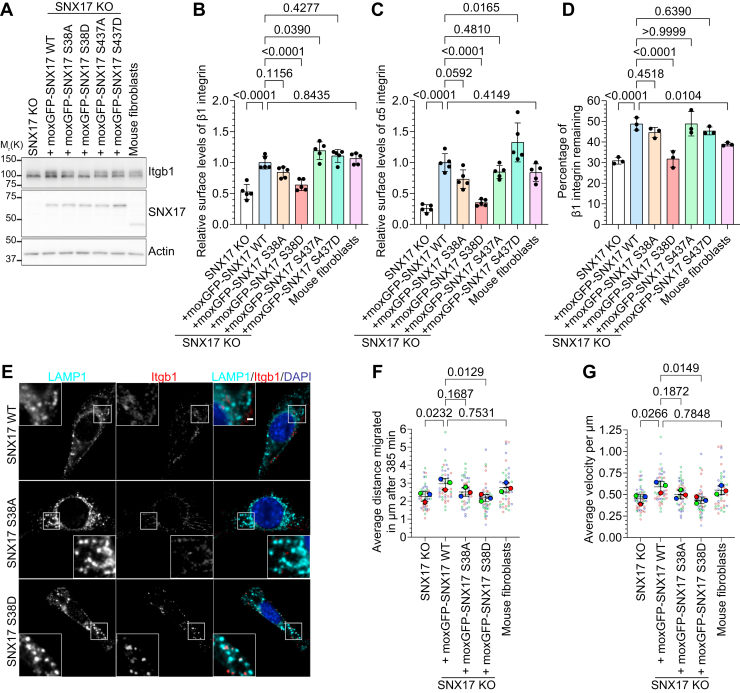
**Phospho-mimicking mutation ofSNX17S38, but not S437, reduces integrin recycling**. *A*, Western blot analysis of WT and SNX17 KO fibroblasts, and SNX17 KO fibroblasts stably re-expressing the indicated moxGFP-tagged SNX17 variants. Actin served as loading control. *B* and *C*, quantification of Itgb1 (*B*) and Itga5 (*C*) surface levels on SNX17 KO fibroblasts re-expressing WT or mutant forms of moxGFP-SNX17 analyzed by flow cytometry (mean ± s.d.; n = 5). Statistical analysis was carried out with one-way ANOVA + Dunnett multiple comparison test compared to surface levels of moxGFP-SNX17 WT-expressing cell line. *D*, degradation of cell-surface β1 integrins within 24 h determined by capture-ELISA in the indicated cell lines (mean ± s.d.; n = 3). Statistical analysis was carried out with one-way ANOVA + Dunnett multiple comparison test compared to moxGFP-SNX17 WT. *E*, localization of endogenous β1 integrin (*red*) after surface labeling with an anti-β1 integrin antibody and internalization for 40 min in SNX17 KO fibroblasts re-expressing moxGFP-SNX17 WT, S38A, or S38D. Cells were fixed and costained with an antibody against LAMP1; nuclei were counterstained with DAPI. Scale bars represent 10 μm; 2 μm for magnified inserts. *F* and *G*, quantification of single cell migrated distance (*F*) and velocity (*G*) of mouse fibroblasts, SNX17 KO cells, and SNX17 KO cells re-expressing moxGFP-SNX17 WT, S38A, or S38D extracted from time-lapse microscopy recordings by single-cell tracking (mean ± s.d of three different experiments with 14 analyzed cells each). Statistical analysis was carried out with one-way ANOVA + Dunnett multiple comparison test compared to moxGFP-SNX17 WT-expressing cell line.

To dissect the specific role of SNX17 S38 and S437 phosphorylation, we generated phosphomimetic (S to D) and nonphosphorylatable (S to A) mutants of both phosphorylation sites. We expressed these mutants and WT moxGFP-SNX17 in SNX17 KO fibroblasts at levels matching endogenous SNX17 (Fig. 2*A* and Fig. S2*F*). While SNX17 S38A, S437A, and S437D expression rescued Itgb1 and Itgα5 surface levels comparable to SNX17 WT, SNX17 S38D expression failed to restore α5β1-integrin surface levels (Fig. 2, *B* and *C*). Surface levels of SNX17-independent cargo proteins Itgb3 (Fig. S2*G*) and ItgαV (Fig. S2*H*) remained unaffected across all SNX17 variants. To investigate how SNX17 S38 and S437 phosphorylation affect cargo protein stability and recycling, we analyzed the degradation kinetics of the surface β1 integrins. Expression of SNX17 WT, S38A, S437A, or S437D rescued β1-integrin stability in SNX17 KO fibroblasts, where β1-integrins typically undergo rapid degradation (Fig. 2*D*). In contrast, SNX17 S38D had no effect, yielding degradation rates like SNX17 KO cells (Fig. 2*D*). Together, these data indicate that S437 phosphorylation mimetics did not affect SNX17-mediated cargo recycling. We therefore focused our subsequent investigations on the S38 phosphorylation site, which showed pronounced effects on SNX17 function when mutated.

To directly visualize SNX17 cargo trafficking in cells, we tracked β1 integrin in SNX17 KO fibroblasts expressing moxGFP-SNX17 WT, S38A, or S38D by antibody-labeling surface β1-integrins before internalization. After 40 min, SNX17 WT and S38A cells showed minimal internalized β1-integrin signal, indicating efficient recycling to the cell surface (Fig. 2*E*). In contrast, β1 integrin accumulated in SNX17 S38D-expressing cells and colocalized with LAMP1-positive lysosomes (Fig. 2*E*), further supporting the observation that SNX17 S38D is nonfunctional and cannot prevent lysosomal targeting of β1 integrin.

Finally, we assessed the functional consequences of SNX17 S38 phosphorylation on cell migration, a process dependent on SNX17 and proper integrin trafficking (29). Random migration assays revealed that cells expressing SNX17 S38D exhibited impaired migration comparable to SNX17 KO cells (Fig. 2, *F* and *G*). The S38A mutation showed reduced, although not statistically significant, migration compared to SNX17 WT-expressing cells. In summary, our findings reveal that SNX17 S38 phosphorylation impairs β1-integrin stability and recycling, resulting in reduced surface levels and compromised cell migration. Conversely, AMPK activation decreases SNX17 protein stability over time but mimicking S437 phosphorylation has no effect on β1-integrin surface levels and stability.

### S38 phosphorylation prevents SNX17 binding to early endosomes

**Figure 3:**
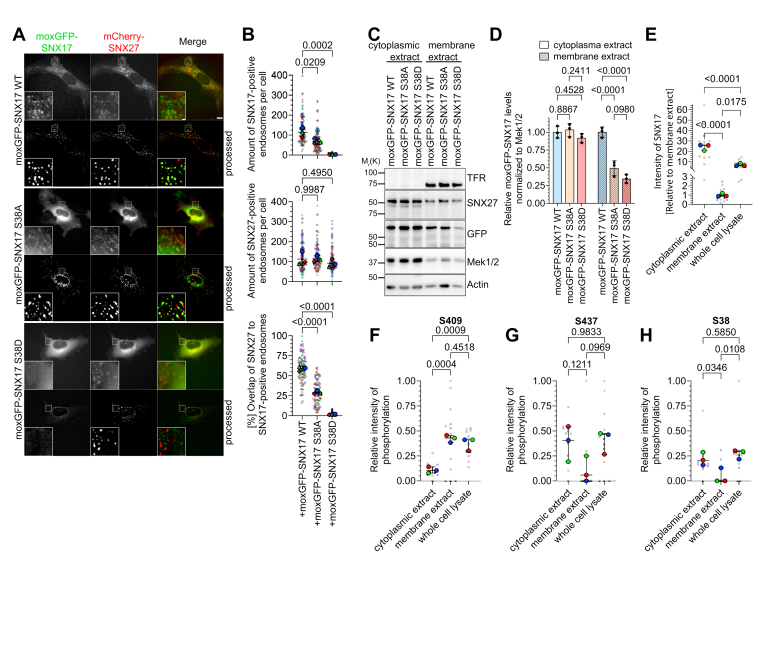
**SNX17 S38Dpreventsbinding to early endosomes**. *A*, representative images of moxGFP-tagged SNX17 (*green*) and mCherry-tagged SNX27 (*red*) in SNX17 fibroblasts expressing moxGFP-SNX17 WT, S38A, or S38D. The fluorescence was determined in living cells on a wide-field fluorescence microscope. Denoised images with background subtraction are marked with “processed”. Scale bars represent 10 μm; 1 μm for zoom area. *B*, quantification of SNX17- and SNX27-positive endosomes as well as the percentage of SNX27/SNX17-double positive endosomes per cell from three different experiments (mean ± s.d; for a total of 88 (WT), 82 (S38A), and 77 (S38D) cells). Statistical analysis was carried out with one-way ANOVA + Dunnett multiple comparison test compared to moxGFP-SNX17 WT-expressing cells. *C*, Western blot analysis of SNX17 KO fibroblasts re-expressing moxGFP-SNX17 WT, S38A, or S38D after fractionation into cytoplasmic and membrane extracts. TFR serves as a marker for the membrane extract. *D*, quantification of moxGFP-SNX17 levels in the indicated extract normalized to Mek1/2 (mean ± s.d.; n = 3). Statistical analysis was carried out with two-way ANOVA + Tukey multiple comparison test. *E*–*H*, relative intensity levels of SNX17 (*E*) and phosphorylated peptides at phosphosites S409 (*F*), S437 (*G*), and S38 (*H*), in whole cell lysates of HeLa SNX17 KO cells stably re-expressing moxGFP-SNX17 WT and after fractionation into cytoplasmic and membrane extracts (mean ± range; n = 3, with 4–5 technical replicates per condition). Statistical analysis was performed using one-way ANOVA followed by Tukey’s multiple comparison test.

Given the correlation between increased cytosolic SNX17 levels and the increased occupancy of S38 phosphorylation (Fig. 1, *G*–*I*), we hypothesized that S38 phosphorylation regulates SNX17 membrane binding. To test this, we analyzed the subcellular localization of moxGFP-tagged SNX17 WT and S38A/D in mouse fibroblasts by live cell microscopy, using mCherry-tagged SNX27 as early endosome marker. While moxGFP-SNX17 S38A maintained punctate endosomal localization and colocalized with mCherry-SNX27, although less prominent compared to moxGFP-SNX17 WT, SNX17 S38D was found exclusively in the cytoplasm (Fig. 3, *A* and *B*). Quantitative analysis of 247 cells revealed an average of 114 ± 24 SNX17-positive endosomes in SNX17 WT cells, 68 ± 13 in S38A cells, and only 3 ± 1 in S38D-expressing cells while the number of SNX27-positive endosomes remained relatively constant across all cell lines (113 ± 33, 114 ± 15, and 92 ± 19, respectively) (Fig. 3*B*). To further confirm this observation, we prepared subcellular fractions of SNX17 KO fibroblasts expressing moxGFP-SNX17 variants. SNX17 S38A and S38D showed reduced levels in the membrane fraction compared to WT, while maintaining similar levels in the cytoplasm (Fig. 3, *C* and *D*). Since these mutants mimic unphosphorylated and phosphorylated states respectively, we hypothesized that membrane-associated SNX17 contains unphosphorylated S38, while cytoplasmic SNX17 contains phosphorylated S38. To test this hypothesis, we used mass spectrometry to analyze the phosphorylation status of SNX17 in membrane and cytosolic fractions isolated from HeLa SNX17 KO cells expressing moxGFP-SNX17 WT. Despite SNX17 being approximately 25-fold more abundant in the cytoplasm than in the membrane extract (Fig. 3*E*), we detected distinct SNX17 phosphorylation patterns in both fractions. S409 phosphorylation was enriched in the membrane extract, while phosphorylated S437 was predominantly detected in the cytoplasm (Fig. 3, *F* and *G*). Importantly, S38 phosphorylation was largely restricted to the cytoplasm, appearing in only two of 13 membrane samples (Fig. 3*H*). These findings support the crucial role of S38 phosphorylation in regulating SNX17 recruitment to early endosomes.

**Figure 4:**
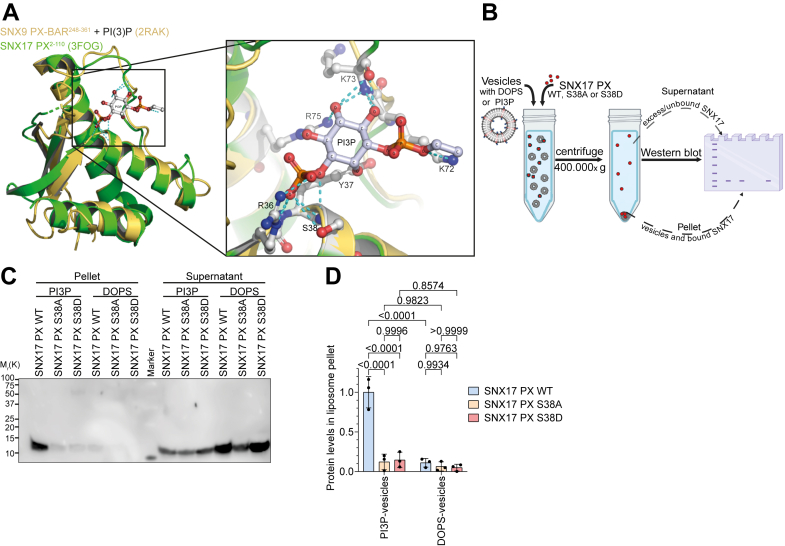
**SNX17 S38Ddoes not bind to PI3P.***A*, structure alignment of the SNX9 PX domain (*yellow*) bound to PI3P (PDB ID: 2RAK) and the PX domain of human SNX17 (*green*; PDB ID: 3FOG). Magnification shows computed hydrogen bonds between PI3P and the PX domain of SNX17. *B*, schematic representation of liposome pelleting assay: Unilaminar DOPC vesicles containing PI3P to mimic endosomes or DOPS as control are incubated with the purified recombinant WT, S38A, or S38D PX domain of SNX17. After ultracentrifugation, the supernatant and the vesicle-rich pellet were analyzed for SNX17 levels by Western blot analysis. *C*, representative Western blot of liposome pelleting assay. *D*, quantification of PX domain binding to liposomes. SNX17 PX WT, S38A, or S38D levels found in pellet fraction are plotted relative to SNX17 PX WT binding to PIP3 vesicles (mean ± s.d.; n = 3). Statistical analysis was carried out with two-way ANOVA + Tukey multiple comparison test compared to SNX17 PX WT.

To understand the structural basis of the interaction between SNX17 S38 and PI3P-containing lipids, we modeled the SNX17 PX domain bound to PI3P based on the known structures of PI3P-bound SNX9 PX-BAR (PDB: 2RAK; Fig. 4*A*) and p40phox (PDB: 1H6H; Fig. S3*A*). Our model predicts hydrogen bonding between the 3′-phosphate group of PI3P, the hydroxyl and amino groups of SNX17 S38, and other nearby amino acids. In addition, the model indicates that S38 phosphorylation would prevent ligand binding by blocking inositol 3-phosphate entry and thereby fulfill the function of a PIP-stop motif similar to Ser72 of SNX3 (34).

To validate the PIP-stop hypothesis and to determine whether S38 phosphorylation directly affects SNX17's ability to bind PI3P-containing membranes, we performed *in vitro* binding assays using recombinant SNX17 PX domains (WT, S38A, and S38D) and large unilamellar vesicles (LUVs) (Fig. 4*B*). To mimic early endosomes, we generated large LUVs containing PI3P and extruding the mixture to produce vesicles of approximately 100 nm in diameter (Fig. S3*B*). As a control, we prepared LUVs containing phosphatidylserine (DOPS), which mimics the single negative charge of PI3P but lacks the specific phosphate group at the 3′ position of the inositol ring. After incubating purified SNX17 PX domain variants with these LUVs, ultracentrifugation and Western blot analysis revealed that WT SNX17 PX domain binding required PI3P, while SNX17 PX S38A and PX S38D mutants lost selective lipid-binding activity (Fig. 4, *C* and *D*). Our findings demonstrate that SNX17 binding to endosomal PI3P requires hydrogen bonds between S38 and the phosphate group of PI3P. Introducing a negative charge at S38 through phosphorylation might inhibit SNX17 binding to endosomal PI3P due to electrostatic repulsion between the negatively charged S38D and the phosphate group of PI3P. This highlights the critical role of S38 phosphorylation in regulating SNX17's membrane association.

### PX domain mutations influence SNX17 binding to cargo proteins and the Retriever complex

**Figure 5:**
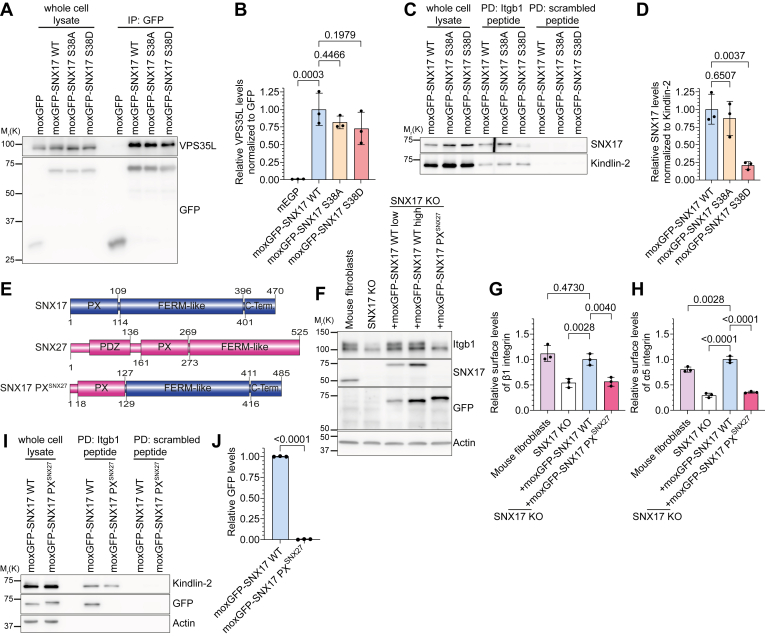
**Mutations in the PX domain of SNX17 influencecargobinding.***A*, Western blot analysis of whole cell lysates and GFP co-immunoprecipitations (IP) of moxGFP and moxGFP-tagged SNX17 variants from HEK293T cells. Blots were probed with antibodies against GFP and VPS35L to determine VPS35L binding. *B*, quantification of co-immunoprecipitated VPS35L normalized to GFP levels and relative to moxGFP-SNX17 WT (mean ± s.d.; n = 3). Statistical analysis was carried out with one-way ANOVA + Dunnett multiple comparison test compared to cells transfected with moxGFP-SNX17 WT. *C*, Western blot of a streptavidin-bead pull-down assay with biotinylated β1-integrin cytoplasmic tail peptides (Itgb1-peptide) or scrambled peptides and cell lysates from moxGFP-SNX17 WT, S38A or S38D cells. After pull-down, bound proteins were detected by Western blotting with antibodies against SNX17 and Kindlin-2. *D*, quantification of moxGFP-SNX17 binding to biotinylated β1-integrin cytoplasmic tail peptides normalized to bound Kindlin-2 (mean ± s.d.; n = 3). Statistical analysis was carried out with one-way ANOVA + Dunnett multiple comparison test compared to moxGFP-SNX17 WT. *E*, schematic domain organization of SNX17, SNX27, and the chimeric protein SNX17 PX^SNX27^, in which the PX domain of SNX17 was replaced by the PX of human SNX27. *F*, Western blot analysis of SNX17 KO fibroblasts re-expressing indicated SNX17 PX variants. Actin served as loading control. *G* and *H*, quantification of surface levels of Itgb1 (G) and Itga5 (H) on SNX17 KO fibroblasts re-expressing indicated SNX17 PX variants analyzed by flow cytometry (mean ± s.d.; n = 3). Statistical analysis was carried out with one-way ANOVA + Dunnett multiple comparison test compared to cells expressing moxGFP-SNX17 WT. *I*, Western blot of streptavidin-bead pull-down assay of biotinylated β1-integrin cytoplasmic tail peptides or scrambled peptides and lysates of SNX17 KO HeLa cells stably expressing moxGFP-SNX17 WT or moxGFP-SNX17 PX^SNX27^. After pull-down, bound proteins were detected by Western blotting with antibodies against GFP (to identify moxGFP-SNX17) and Kindlin-2. *J*, quantification of moxGFP-SNX17 binding to biotinylated β1-integrin cytoplasmic tail peptides (mean ± s.d.; n = 3). Statistical analysis was carried out with unpaired *t* test (two-tailed) with 95% confidence interval.

Finally, we investigated whether S38 phosphorylation only inhibits SNX17's membrane interaction or whether it also affects cargo protein and Retriever complex binding. Consistent with published data, immunoprecipitation (IP) of moxGFP-SNX17 WT detected VPS35L, a component of the Retriever complex (Fig. 5, *A* and *B*). Both SNX17 S38A and S38D mutants maintained VPS35L interaction, though at slightly reduced levels (Fig. 5, *A* and *B*). To assess how SNX17 S38 phosphorylation affects cargo binding, we performed pull-down assays using synthetic peptides representing WT β1-integrin cytoplasmic domains or scrambled peptides. These peptides were incubated with cell lysates containing moxGFP-tagged SNX17 WT, SNX17 S38A, or S38D. WT SNX17 and the known integrin interactor Kindlin-2 specifically bound the β1-integrin tail peptide, but not the scrambled control. Importantly, the SNX17 S38A mutant maintained this cargo-binding interaction, whereas S38D exhibited significantly reduced binding (Fig. 5, *C* and *D*). These findings suggest that modifications to the SNX17 PX domain, such as Ser38 phosphorylation, can impact its ability to recognize and bind cargo proteins.

To determine whether this reduced cargo binding stemmed from impaired PI3P binding or broader PX domain effects, we created a chimeric construct by replacing the PX domain of SNX17 with that of human SNX27 (Fig. 5*E*). The PX domain of SNX27 also binds to PI3P-containing LUVs *in vitro* (Fig. S4, *A* and *B*) and PI3P-positive endosomes in cells (35) but lacks a phosphorylation site corresponding to S38 in SNX17. We stably expressed the moxGFP-tagged chimeric construct SNX17 PX^SNX27^ in SNX17 KO fibroblasts at expression levels similar to moxGFP-SNX17 WT (Fig. 5*F* and Fig. S4*C*). This protein is no longer recognized by the commercial anti-SNX17 antibody (Fig. 5*F*). The chimeric moxGFP-SNX17 PX^SNX27^ maintained endosomal localization and colocalized with mScarlet-SNX17 WT (Fig. S4*D*) or mCherry-SNX27 (Fig. S4*E*). However, the chimeric moxGFP-SNX17 PX^SNX27^ failed to rescue cell surface levels of β1 integrin (Fig. 5*G*) and Itga5 (Fig. 5*H*), indicating that it was functionally inactive. Given that the chimeric SNX17 PX^SNX27^ maintained its membrane association, we sought to study the underlying cause of its functional inactivity. To this end, we investigated the chimera's capacity to interact with two key partners: the β1-integrin cargo protein and the Retriever complex. The exchange of the PX domains between SNX17 and SNX27 enhanced the interaction of moxGFP–SNX17 PX^SNX27^ with the Retriever subunit VPS35L in HeLa cells (Fig. S4, *F* and *G*). In contrast, binding to β1-integrin tail peptides was completely inhibited (Fig. 5, *I* and *J*). This suggests that the SNX17 PX domain plays a role in cargo binding that extends beyond simply facilitating membrane-targeting. This implies a complex interplay between SNX17's endosomal localization, cargo recognition, and Retriever complex interaction, with the PX domain serving as a key modulator of these functions.

## Discussion

SNXs comprise a diverse protein family crucial for intracellular protein trafficking and sorting. These proteins recognize specific lipid environments through their phosphoinositide-binding PX domains and facilitate transport of cargo proteins between cellular compartments (36, 37). SNX17 specifically binds directly to cargo proteins *via* its FERM domain, linking them to the Retriever complex for recycling (6). Our findings reveal a complex interplay between SNX17's endosomal localization, cargo recognition, and Retriever complex interaction, with the PX domain serving as a key modulator of these functions. The S38 phosphorylation site within the PX domain acts as a molecular switch, regulating membrane association of SNX17 through a mechanism reminiscent of PIP-stop motifs. This phosphorylation not only affects PI3P binding but also influences cargo interaction, suggesting an expanded role for the SNX17 PX domain beyond mere lipid binding.

To address concerns about potential adverse effects of high SNX17 expression levels on endosomal protein trafficking, we analyzed SNX17 activity and membrane recruitment in mouse fibroblasts and HeLa cells with increasing SNX17 levels. Surprisingly, increased SNX17 expression did not significantly impact the endosomal trafficking of β1 integrins or SNX17-independent cargo proteins. Moreover, we observed that the amount of membrane-localized SNX17 remained constant despite increasing cytosolic levels, suggesting a regulatory mechanism to maintain homeostasis. In search of a mechanistic explanation for increased cytosolic SNX17 levels, our investigation revealed that SNX17 S38 phosphorylation increased concomitantly with SNX17 expression. Structural modeling predicted that S38 phosphorylation prevents recruitment to endosomes by blocking PI3P binding, as the negative charges from the phosphorylation and the phosphate of PI3P repel each other. This prediction was corroborated by our experimental findings: a phospho-mimicking mutation abolished PI3P binding *in vitro*, dislodged SNX17 from endosomes, and inactivated SNX17 function in cells.

Our findings on SNX17 S38 phosphorylation align with previously described PIP-stop motifs in other SNX proteins (38, 39), such as SNX3 S72, SNX1, and SNX12 (34, 40). This suggests a broadly conserved mechanism for rapid and reversible control of protein–membrane interactions and cargo sorting. Subcellular fractionation studies provide strong evidence for this phosphorylation-dependent localization model: the phospho-mimicking SNX17 S38D mutant localizes exclusively to the cytosol, and mass spectrometry analysis demonstrates that S38 phosphorylation is barely detectable in membrane fractions while present in cytosolic extracts. Corroborating these findings, proteomic analyses have detected phosphorylated SNX3 S72 and SNX12 S73 only in cytosolic fractions, whereas their nonphosphorylated counterparts associate with membrane-bound organelles (41). Remarkably, of the 51 human PX domain-containing proteins, 26 possess a serine or threonine at positions corresponding to SNX17 S38 or SNX3 S72 (38, 42), suggesting widespread potential for this regulatory mechanism. Spatial proteomics data from human and mouse cells further reinforce this trend, revealing that SNX family members with a potential phosphorylation site in the conserved lipid-binding pocket are more abundant in the cytoplasm compared to those lacking this site (43, 44). These observations lead us to propose that PIP-stop phosphorylation maintains a dynamic equilibrium between membrane-bound and cytosolic SNX17, potentially preserving the homeostasis of cargo protein trafficking even under conditions of SNX17 overexpression. This mechanism may prevent undue competition with other PI3P-binding proteins, ensuring balanced membrane recruitment across various trafficking pathways.

The precise regulation of PIP-stop motif phosphorylation in SNXs remains an active area of investigation. While previous studies suggested Jak1/2, PAK1, PKA, and PKC family members as potential kinases (38), our experiments did not detect increased SNX17 S38 phosphorylation upon AMPK or PKC activation (Speidel & Böttcher, unpublished). Our comprehensive *in silico* kinase library screen for SNX17 S38 phosphorylation has expanded the list of candidates to include SNRK, DAPK2, DAPK1, and kinases of the CAMK class (45, 46). Intriguingly, increased phosphorylation of SNX3 and SNX17 PIP-stop motifs has been observed during cold ischemia, coinciding with MAPK stress-response pathway activation (47). Moreover, phosphorylation at corresponding sites in SNX1, SNX3, SNX12, and SNX17 has been reported in various cancer types (47, 48, 49, 50, 51, 52), hinting at a potential link between SNX phosphorylation and disease states.

Our study reveals novel insights into the role of the SNX17 PX domain in cargo binding beyond the established membrane-targeting role, particularly in relation to β1 integrin. While the PX domain's primary function is phosphoinositide binding, previous studies have shown that PX domains are also capable of protein–protein interactions (53, 54). We demonstrated that phosphorylation of SNX17 at S38 reduces its binding to β1 integrin, suggesting a potential mechanism for modulating SNX17 activity. The chimeric SNX17 PX^SNX27^ construct further emphasized the critical nature of the PX domain in cargo binding, independent of its endosomal localization function. Intriguingly, this construct maintained endosomal localization but failed to rescue β1-integrin surface levels indicating that the PX domain's function in cargo recognition encompasses more than its known role in lipid binding. The interplay between the PX and FERM domains in SNX17 appears to be intricate. A mutation in the SNX17 FERM-like domain (V380D) inhibits both cargo binding and endosomal recruitment (16), while our data suggest the PX domain exerts an inhibitory effect on FERM domain cargo binding. Though the precise mechanism remains unclear and warrants further investigation, recent studies support a functional interplay between different SNX17 domains. These studies reveal that SNX17 exists in an autoinhibited state where part of the C-terminal tail competitively blocks the cargo recognition site within the FERM domain (16, 17, 55). SNX17 binding to PI3P facilitates its membrane attachment, which releases this autoinhibition, exposes the Retriever-binding motif for interaction with the Retriever complex, and liberates the cargo recognition site (55). Our results align with this model, as the phospho-mimicking SNX17 S38D mutant failed to bind PI3P, thereby remaining in its autoinhibited form characterized by reduced cargo and Retriever binding. Taken together, our findings support a model in which SNX17 exists in an autoinhibited form, with its activity finely regulated by phosphorylation and intramolecular interactions. This mechanism enables dynamic control of SNX17 function, allowing cells to adjust endosomal sorting processes in response to changing cellular conditions.

## Experimental procedures

### Antibodies and reagents

The following antibodies were used for Western blotting (WB) or immunofluorescence (IF): anti-SNX17 (Proteintech; 10275-1-AP; WB 1:1500); anti-β-Actin (Sigma-Aldrich; A5441; WB 1:2000); anti-GFP (Sigma-Aldrich; 11814460001; WB 1:1000); anti-Integrin β1 CD29 [Clone 18] (BD Transduction Laboratories; 610,468; WB 1:1000); anti-VPS35L (Invitrogen; PA5-28553; WB 1:1000); anti-pS79 ACC1 (Cell Signaling; 3661; WB 1:1000); anti-Mek1/2 (Cell Signaling; 9122; WB 1:1000); anti-Kindlin-2 (Millipore; MAB2617; WB 1:1000); anti-SNX27 (Bethyl Laboratories; A305-439A; WB 1:2000); anti-Transferrin Receptor [Clone H68.4] (Invitrogen; 13-6800; 1:1000); and anti-GAPDH (Calbiochem; CB1001; WB 1:10,000). Anti-mouse integrin β1 antibody (WB 1:10,000; IF 1:200) was home-made (56). The following antibodies were used for flow cytometry: anti-mouse αV-integrin PE (551187; BD Pharmingen; 1:300), anti-mouse α5-integrin PE (557447; BD Biosciences; 1:300), anti-mouse α5-integrin A647 (553319; BD Pharmingen; 1:300), anti-mouse β1-integrin PE (557416; BioLegend; 1:300), anti-mouse β3-integrin PE (12-0611; 2C9.G3; eBioscience; 1:300), and anti-TFR Biotin (BD; GoH3; BD Biosciences; 1:300). Cy-5 Streptavidin (016-170-084; Jackson; 1:500) was used as secondary antibody for anti-TFR Biotin. As isotype controls hamster IgG PE (eBio299Arm, eBioscience, 1:300) for anti-mouse β1-integrin PE and anti-mouse β3-integrin PE, rat IgG1 PE (554685; R3-34; PharMingen; 1:300) for anti-mouse αV-integrin PE, rat IgG2a APC (400512; BioLegend; 1:300) for anti-mouse α5-integrin A647, rat IgG2a biotin (13-4321, eBioscience) for anti-TFR Biotin, and rat IgG2a PE (555844; R35-95; PharMingen; 1:300) for anti-mouse α5-integrin, PE were used. The following chemicals were used: protease inhibitor cocktail (Roche; 4693159001); A-769662 (LC Lab; A-1803); DMSO (042,780.AK, Thermo Fisher Scientific); phosphatase inhibitor cocktail 2 & 3 (Sigma-Aldrich; P5726 & P0044); and L-Glutathione (Sigma-Aldrich; G4251). The following antibodies were used for immunofluorescence: anti-LAMP1 (DSHB; 1D4B; IF 1:400), goat anti-rat IgG (H+L) Alexa Fluor 647 (Invitrogen; A-21247; 1:500), and donkey anti-rabbit IgG (H+L) Cy3 (Jackson ImmunoResearch; 711-165-152; 1:800).

### Cell lines

SV40 large T-immortalized mouse kidney fibroblasts (29), HeLa cells (American Type Culture Collection, CCL-2), and HEK293T cells (ATCC, CRL-3216) were cultured in Dulbecco's modified Eagle's medium (DMEM; Gibco) with 10% fetal bovine serum (FBS; v/v; Gibco) and 1% penicillin–streptomycin (P/S; v/v; Gibco) at 37 °C, 5% CO_2_, and 95% humidity. U2OS human osteosarcoma were cultured in McCoy’s 5a Medium (Invitrogen) supplemented with 10% FBS, 1% glutamine, and 1% P/S. To generate HeLa SNX17 KO, cells were transiently transfected with CRISPR-hSpCas9 vectors that possess SNX17 guide-RNAs. After 48 h selection with 2 μg/ml puromycin, cells were FACS-sorted for single-cell clones and for low α5-integrin surface levels to enrich SNX17 KO clones. SNX17 KO clones were confirmed by WB analysis. SNX17 KO mouse kidney fibroblasts were described before (57). All cell lines were regularly checked for *mycoplasma*.

### Constructs and plasmids

Point mutations into the mouse SNX17 PX and FERM domains (S38A, S38D, S437A, S437D) were introduced by site-directed mutagenesis; the chimeric SNX17/SNX27 variant was cloned *via* Gibson assembly. SNX17 as well as SNX27 cDNAs were cloned in frame with N-terminal moxGFP or mScarlet cDNA into the retroviral expression vector pRetroQ by Gibson assembly or restriction enzyme digestion. GST-SNX17 PX WT, S38A, and S38D were cloned into pGEX-4T-1 vector by extension PCR and restriction enzyme digestion. WT GST-SNX27 PX was cloned into pGEX-4T-1 vector by Gibson assembly. The following primers were used:

**Table:** 

SNX17_S38 A_fwd.	GCCCCAGAAGCTGGGcgTAGCGCACCCGAC
SNX17_S38 A_rev.	GTCGGGTGCGCTACgcCCAGCTTCTGGGGC
SNX17_S38D_fwd.	GCCCCAGAAGCTGGtcGTAGCGCACCCGAC
SNX17_S38D_rev.	GTCGGGTGCGCTACgaCCAGCTTCTGGGGC
SNX17_S437 A_fwd.	GTGAAACTCTCAAGTAAACTGgcTGCTGTGAGCTTGCGGGGG
SNX17_S437 A_rev.	CCCCCGCAAGCTCACAGCAgcCAGTTTACTTGAGAGTTTCAC
SNX17_S437D_fwd.	GTGAAACTCTCAAGTAAACTGgaTGCTGTGAGCTTGCGGGGG
SNX17_S437D_rev.	CCCCCGCAAGCTCACAGCAtcCAGTTTACTTGAGAGTTTCAC
PX_mSNX17_vector_fwd.	agaactacCCCACAGAGGAGGTTTCCTTGG
PX_mSNX17_vector_rev.	tgggatctagcttgtacagctcgtccatgcc
PX_hSNX27_fragment_fwd.	ctgtacaagctagatcccagtgacgactcg
PX_hSNX27_fragment_rev	CTCTGTGGGgtagttctcatcggattctgataggaat
TEV-reco. SNX17 fwd.	ggcacgaaGAAAATTTATATTTTCAAggcATGCACTTTTCCATTCCTGAAACG
Avi-reco. SNX17 rev.	ggcacgaaGAAAATTTATATTTTCAAggcATGCACTTTTCCATTCCTGAAACG
TEV-reco. SNX27 fwd.	agaactacTAAGCGGCCGCATCGTG
Avi-reco. SNX27 rev.	gggatctaggccTTGAAAATATAAATTTTCttcgtgcca
reco. SNX27 insert fwd.	TTCAAggcctagatcccagtgacgactcg
reco. SNX27 insert rev.	GGCCGCTTAgtagttctcatcggattctgataggaat

For CRISPR-mediated depletion of human SNX17, the guide-RNA 5′-gcgggactcggtttcgggaa-3′ was ligated into pSpCas9(BB)-2A-Puro vector V2.0 (gift from Feng Zhang (Addgene plasmid # 62988; http://n2t.net/addgene:62988; RRID:Addgene_62988) (58)). The correct sequences of all constructs were verified by Sanger sequencing (Microsynth Seqlab).

### Transient transfection and stable viral transduction

Cells were transiently transfected with Lipofectamine 2000 (Invitrogen) according to the manufacturer's protocol. To generate stable cell lines, HEK293T cells were transiently transfected with vesicular stomatitis virus G glycoprotein–pseudotyped retroviral vectors. Produced viral particles were concentrated from cell culture supernatant as previously described (30) and 5 to 10 μl were used for the infection of 60,000 cells seeded in 6-well plates the day before. Infected cells were selected with 2 μg/ml puromycin for 3 days and further sorted to different expression levels by FACS.

### Flow cytometry

Cells were grown overnight to 80% confluency, trypsinized, and centrifuged for 5 min, 300×*g*. Cell pellets were washed with PBS, resuspended in FACS buffer (1% BSA, 5 mM EDTA in PBS), and 200,000 cells were incubated for 30 min with the corresponding antibody dilution at 4 °C. After washing with FACS buffer, the GFP intensities and surface levels of integrins of at least 10,000 cells were measured on BD LSRFortessa X-20 Cell Analyzer (BD Biosciences) equipped with FACSDiva software (BD Biosciences, version 9.0). FlowJo software (BD Biosciences, version v10.10.0) was used to analyze the geometric mean of the fluorescence intensity of each sample. Sorting of stable cell lines according to their GFP levels was done analogously on a BD FACSAria III.

### Immunofluorescence of internalized integrin, live cell imaging, and image analysis

To analyze internalized integrin, 5000 cells were cultured on a μ-Slide 8 Well (ibidi, 80827) coated with 5 μg/ml fibronectin (Calbiochem) for 3.5 h at 37 °C, washed with PBS, and subsequently incubated with a homemade anti-mouse β1-integrin antibody (1:200 in PBS) for 30 min at 4 °C. After two times washing with PBS, the surface-bound antibody was allowed to internalize in DMEM + 5% FBS + 1% P/S for 40 min at 37 °C. Remaining antibody at the cell surface was stripped by washing the cells with PBS and two times with an acidic glycine buffer (150 mM NaCl; 1 mM MgCl_2_; 0,125 mM KCl_2_; 100 mM Glycine (pH 2.5)). Subsequently, cells were fixed with 4% PFA/PBS for 10 min at room temperature (RT), permeabilized for 4 min with 0.1% Triton/PBS, blocked for 2 h with blocking solution (5% BSA/PBS) at 4 °C, and incubated overnight with an anti-LAMP1 antibody. Cells were washed three times with blocking solution and incubated with secondary antibodies for 2 h. DNA was counterstained with DAPI (Sigma) for 10 min. To analyze the localization of moxGFP-SNX17 in fixed cells, cells were fixed, permeabilized, and stained as described above. Images were captured using a LSM780 confocal laser scanning microscope (Zeiss) with a 40x/1.4 oil objective *via* the ZEN software (black version).

For live-cell-imaging, 10,000 cells were cultivated on μ-Slide 8 Well (ibidi, 80827) coated with 5 μg/ml fibronectin overnight. The medium was changed to Phenol-red-free DMEM (Gibco; 21063-029) supplemented with 10% FBS, and cells were imaged as 3D stacks live on a widefield Leica Thunder microscope using a 63x HCX PL APO 63x/1.20 W CORR λ BL objective at 37 °C and 5% CO_2_ supply. In some experiments, cell nuclei were stained using Hoechst 33342 (Miltenyi Biotec; 130-111-569). Before image analysis, images were subjected to Gaussian filter (radius of 7 sigma) with Fiji software (59), subtracted from the original images, and subsequently deconvolved with Huygens Professional (version 23.10.0p7) with "Classic MLE" as the deconvolution algorithm parameter (Scientific Volume Imaging, The Netherlands, http://svi.nl). To calculate the number of endosomes, a threshold was set manually for the signal intensity on the endosomes of each cell. Endosomes were identified through the MorphoLibJ plugin (60), utilizing the function Binary Images Connected Components Labeling (connectivity = 26; type = 16 bits), and quantified by the number of binary levels.

### Integrin stability assay and capture ELSIA

250,000 cells were seeded on 6-well plates coated with 5 μg/ml fibronectin the day before the experiment. Cells were washed twice with cold PBS and cell surface proteins were biotinylated with 1 ml of a 0.2 mg/ml Sulfo-NHS-LC-Biotin (Thermo Fisher Scientific; 21335) dilution in PBS for 45 min on ice. After two washes with cold PBS, cells were either lysed with 300 μl ELISA-lysis buffer (200 mM NaCl, 75 mM Tris (pH 7.5), 15 mM NaF, 1.5 mM Na3VO4, 7.5 mM EDTA and 7.5 mM EGTA, 1.5% Triton X-100, 0.75% Igepal CA-630 complemented with protease inhibitor cocktail) for 10 min on ice (0 h time point) or cultured in DMEM containing 0.2% FBS, 1% P/S for 24 h at 37 °C (24 h time point) before lysis. Samples were sonicated and centrifuged with 14,000 rpm for 10 min at 4 °C. For capture ELISA, Maxisorb 96-well plates (Thermo Fisher Scientific; 442404) were coated with 50 μl anti-Itgb1 antibody (Millipore; mAB1997; 1:200) in 0.05 M Na_2_CO_3_ buffer (pH 9.6) at 4 °C overnight. Before adding the cell lysate, unspecific binding was blocked with 5% BSA in PBS/0.1% Tween-20 (PBS-T) for 2 h at RT and washed twice with PBS-T. 100 μl lysate of each sample were incubated at 4 °C overnight. Wells were washed 3x with PBS-T and incubated with 1:1500 Streptavidin-HRP (Jackson; 016-030-084) in 1% BSA/PBS-T for 1 h at 4 °C. After washing again three times with PBS-T, 1-Step ABTS (Thermo Fisher Scientific, 37615) was added to measure the amount of biotinylated β1 integrin using a microplate reader at 405 nm (Spectramax ABS Plus, Molecular Devices; 736-0917). For the determination of Itgb1 stability after A-769662 treatment, the cells were prestimulated with A-769662 for 2 h before labeling with Sulfo-NHS-LC-Biotin and further stimulated in DMEM + 1% FBS + 1% P/S for 24 h.

### Random migration assay

10,000 cells were seeded on 5 μg/ml fibronectin-coated 6-well plates for 8 h. Subsequently, plates were placed within an incubation chamber of EVO FL Auto 2 microscope (Invitrogen; AMAFD2000) at 37 °C and 5% CO_2_. The images were captured with a 10× EVOS 9.2 objective (AMEP4681) every 5 min for 24 h. At least six image fields of view were captured per well. For the analysis, single, nondividing cells were manually tracked after 1 h in the incubation chamber for the next 385 min using the MTrackJ plugin (61) in the Fiji ImageJ software.

### Recombinant proteins

GST-SNX17 PX WT, S38A, and S38D as well as WT GST-SNX27 PX were expressed in *Escherichia coli* Rosetta at 18 °C overnight. Bacteria pellets were lysed in high-salt sodium phosphate buffer (200 mM NaCl, 10 mM Na_2_HPO_4_, 5 mM EDTA, pH 7.4, and 1 mM Tris(2-carboxyethyl)phosphine) and purified on EconoFit Profinity GST Columns (Biorad; 12009295). The eluates were dialyzed with a SnakeSkin Dialysis Tubing (Thermo Fisher Scientific; 68100; 10K MWCO) in Hepes buffer (20 mM Hepes; 150 mM NaCl (pH 7.4)) and the tag was removed by His-Tev3 Protease Protease (obtained from the in-house facility) digestion overnight at 4 °C. Subsequently, the protein solutions were concentrated with an Amicon Ultra cutoff 10 kDa (Merck; UFC901024), filtered with Ultrafree-MC, GV 0.22 μm (UFC30GVNB, Merck), and further purified by SEC Superdex 75 Increase (Sigma, 29148721) using Hepes buffer.

### LUV preparation and pelleting

To generate PIP3-containing LUVs, 18:1 1,2-dioleoyl-sn-glycero-3-phospho-(1′-myo-inositol-3′-phosphate) (PI3P; Avanti; 850150) was protonated as described before (20). Briefly, PI3P powder was resuspended in chloroform (CHCl_3_), dried under nitrogen (N_2_) for 1 h in a vacuum pump, and then incubated in a mixture of CHCl_3_:MeOH:1N HCl in a 2:1:0.01 M ratio at RT for 15 min. Lipid solution was dried again under N_2_, resuspended in CHCl_3_:MeOH in a 3:1 ratio, and subsequently dried once again under N_2_. Finally, dried PI3P as well as 1,2-dioleoyl-sn-glycero-3-phosphocholine (DOPC; Avanti; 850375), 1,2-dioleoyl-sn-glycero-3-phospho-L-serine (DOPS; Avanti, 840035P), ATTO655-labeled 1,2-dioleoyl-sn-glycero-3-phosphoethanolamine (Atto655-DOPE; Atto-Tec; AD 655-161) were dissolved in CHCl_3_ and stored at −20 °C. For PI3P^+^-LUVs, DOPC, DOPS, PI3P, and Atto655-DOPE were mixed at a molecular ratio of 80:10:10:1; for DOPS^+^-LUVs, DOPC, DOPS, and Atto655-DOPE were mixed at a molecular ratio of 80:20:1. Lipid solutions were evaporated under N_2_ for 30 min, dried overnight in a vacuum pump, and dissolved in 400 μl Hepes buffer (20 mM Hepes; 150 mM NaCl (pH 7.4)) to a concentration of 1.31 mM. The resulting cloudy lipid solution was frozen and thawed seven times with liquid nitrogen to obtain unilamellar liposomes and then homogenized 31 times in an extruder (Avestin) using a 100 nm membrane (Whatman Nuclepore PC 19 mm; 800309) between two Whatman drain disc (230300). Homogeneous LUV solutions were analyzed for size with the DLS instrument (DynaProNanoStar). For interaction with SNX17, recombinant SNX17 PX WT, S38A, and S38D were added to LUV solutions diluted with Hepes buffer (0.5 mM Hepes; 150 mM NaCl (pH 7.4)) to a final concentration of 20 μM. The mixtures were incubated for 30 min at 37 °C and then ultracentrifuged at 400,000xg for 30 min at 4 °C. The supernatants were mixed 1:1 (v/v) with 4x Laemmli buffer. The Atto655-labeled, pelleted liposomes were washed twice with Hepes buffer, ultracentrifuged again, and finally resuspended in a mixture of Hepes buffer and 4x Laemmli buffer at a ratio of 1:1 (v/v). Samples were loaded onto a 10% Tricin gel and analyzed by Western blotting.

### Cell fraction

Cytoplasmic and membrane extracts were prepared using the Subcellular Protein Fractionation Kit (Thermo Fisher Scientific; 78840). Briefly, trypsinized cells were pelleted (500×*g*, 5 min), washed with ice-cold PBS, and centrifuged again. For cytoplasmic extraction, the pellet was resuspended in cytoplasmic extraction buffer with 5% protease inhibitor cocktail and 1% phosphatase inhibitor 2 & 3 (Roche; 04693159001), incubated for 10 min at 4 °C with gentle mixing, and centrifuged (500×*g*, 5 min). The supernatant (cytoplasmic extract) was collected. To improve the purity of the membrane fraction, the remaining pellet was washed with 900 μl PBS, centrifuged at 500×*g* for 5 min, and resuspended in ice-cold membrane extraction buffer containing 5% protease inhibitor cocktail and 1% phosphatase inhibitor 2 & 3 incubated for 10 min at 4 °C and centrifuged (3000×*g*, 5 min) to obtain the membrane extract. For whole-cell lysate control, cells were lysed in lysis buffer (50 mM Tris–HCl, pH 7.5; 150 mM NaCl; 1 mM EDTA; 1% Triton X-100; 0.1% sodium deoxycholate (SDC)), supplemented with 5% protease inhibitor cocktail and 1% phosphatase inhibitor 2 & 3.

### Peptide pulldown (PD) and IP

Cells were lysed in lysis buffer (50 mM Tris–HCl (pH 7.5), 150 mM NaCl, 1 mM EDTA, 1% Triton X100, and 0.1% SDC supplemented complemented with 5% protease inhibitor cocktail (Roche; 04693159001) and, if phosphorylation was involved, 1% phosphatase inhibitors 2 & 3 (Sigma-Aldrich; P5726P and P0044)). To analyze SNX17 protein interactions, cells were lysed in Mammalian Protein Extraction Reagent (M-PER buffer, Thermo Fisher Scientific, 78501), sonicated, and centrifuged with 14,000 rpm for 10 min at 4 °C. The supernatant was loaded onto 8% SDS-PAGEs, 10% Tricin gels, or used for peptide pulldown or GFP-based IPs.

For peptide pulldown, N-terminally desthiobiotinylated peptides (integrin β1 wt cytoplasmic tail 758–798: HDRREFAKFEKEKMNAKWDTGENPIYKSAVTTVVNPKYEGK-OH; scrambled peptide EYEFEPDKVDTGAKGTKMAKNEKKFRNYTVHNIWESRKVAP-OH) were immobilized on 25 μl Dynabeads MyOne Streptavidin C1 (10 mg/ml, Invitrogen) for 4 h as described before (62). After washing 3x with M-PER buffer, peptide-loaded Dynabeads were incubated with 0.4 mg cell lysates overnight at 4 °C, washed 3x with M-PER buffer, and boiled in 4x Laemmli buffer at 95 °C for 6 min. The eluted fraction was separated on an 8% SDS–PAGE and followed by Western blotting or MS analysis.

For GFP-IPs, 50 μl GFP nanotrap beads (Chromotek) were washed 3x with lysis buffer and incubated with lysate for a minimum of 1 h or overnight at 4 °C. Washing and elution was performed as described above using M-PER buffer.

### Protein detection by Western blotting and Coomassie staining

Proteins in gels were stained using Quick Coomassie Stain (Serva; 35081.01). For Western blotting, proteins were transferred to 0.2 μm nitrocellulose membranes using Trans-Blot Turbo transfer packs (Bio-Rad; 1704270) and a Trans-Blot Turbo system (Bio-Rad; 1704150). After transfer, membranes were blocked for 1 h with 3% BSA/TBS containing TBS-T at RT, then incubated with the primary antibody solution overnight at 4 °C. After three TBS-T washes, membranes were incubated with the secondary antibody for 1 h at RT, washed again three times with TBS-T, and treated with Immobilon Western HRP Substrate (Merck; WBKLS0500). Stained gels and chemiluminescent signals from membranes were captured using an Amersham Imager 600 (GE Healthcare).

### LC–MS/MS data acquisition and data analysis

Sample-loaded GFP nanotrap beads were incubated at 37 °C for 20 min with SDC buffer (50 μl, containing 1% SDC from Sigma-Aldrich, 40 mM 2-chloroacetamide from Sigma-Aldrich, and 10 mM Tris(2-carboxyethyl)phosphine from Thermo Fisher Scientific in 100 mM Tris, pH 8.0). Next, the samples were diluted with 50 μl of milliQ water, and the proteins were digested overnight at 37 °C by adding 0.5 μg of trypsin (Promega). The resulting supernatant was collected using a magnetic rack and acidified with TFA (TFA; Merck) to a final concentration of 1%. Any precipitated SDC was removed *via* centrifugation, and the peptide mixture was desalted using SCX StageTips. Following elution, the samples were vacuum dried and reconstituted in 12 μl of buffer A (0.1% formic acid). Finally, 2 μl of each sample were injected into the LC-MS system.

The LC-MS system comprised an Easy-nLC 1200 (Thermo Fisher Scientific) coupled to an Orbitrap Exploris 480 mass spectrometer (Thermo Fisher Scientific). Desalted peptides were loaded onto a 60 °C-heated reverse-phase column (30 cm, inner diameter: 75 microns; packed in-house with ReproSil-Pur C18-AQ 1.9-micron beads from Dr Maisch GmbH). Peptides were initially loaded in buffer A (0.1% formic acid) on the LC-system and separated through the column at a flow rate of 300 nl/min by a gradient of increasing percentages of buffer B (80% acetonitrile, 0.1% formic acid) as follows: a linear increase from 5% to 30% buffer B over 45 min, followed by a stepwise increase to 65% buffer B over 5 min, then to 95% over the next 5 min. The percentage of buffer B was held at 95% for an additional 5 min.

The mass spectrometer was operated in a data-dependent mode with survey scans ranging from 300 to 1650 m/z (with a resolution of 60,000 at m/z = 200). Up to 15 of the most intense precursor ions were selected for fragmentation using higher energy collisional dissociation with a normalized collision energy of 28. The MS/MS spectra were recorded at a resolution of 15,000 (at m/z = 200). The automatic gain control target for both MS and MS/MS scans was set to 3E6 and 1E5, respectively, with a maximum injection time set to “auto.” Dynamic exclusion was set to 30 s.

Raw data were processed using the MaxQuant computational platform (version 2.2.0.0) (63) with standard settings applied. Shortly, the peak list was searched against the Uniprot database of *Homo sapiens* including mouse SNX17 and GFP with an allowed precursor mass deviation of 4.5 ppm and an allowed fragment mass deviation of 20 ppm. Cysteine carbamidomethylation was set as static modification; methionine oxidation and N-terminal acetylation were set as variable modifications. The match between the run option was enabled, and proteins were quantified across samples using the label-free quantification algorithm in MaxQuant as LFQ intensities. The mass spectrometry proteomics data have been deposited to the ProteomeXchange Consortium *via* the PRIDE (64) partner repository with the dataset identifier PXD061808.

To calculate the relative occupancy of the phosphorylation, the intensities of the phosphorylated peptides were divided by the intensities of the nonphosphorylated peptides. The resulting values were then calculated relative to the mean value of all divisions of the respective experiment and finally normalized to 1 by dividing by the maximum value. To calculate the relative intensity of phosphorylation, the intensities of the phosphorylated peptides were divided by the LFQ intensities of SNX17 for each sample and normalized to the median of the lowest SNX17 levels, which were set to 1. For fractionation experiments, the intensities of the phosphorylated peptides in each fraction were divided by the corresponding SNX17 intensities for each sample and then normalized by dividing by the maximum relative intensity measured for the indicated phosphorylated peptide. Samples in which the sequence coverage of SNX17 was below 16% were excluded from the calculation.

### Structural and statistical analysis

Alignments of protein structures were performed with PyMol Molecular Graphics System (version 2.5.4). Quantitative analysis of gels/blots was performed using Image Studio Lite 5.2.5, normalized to the mean of all quantified signals per experiment. The statistical analysis was carried out using GraphPad Prism software (version 10.2.2). Results are expressed as mean ± sd and statistical significance was determined using one-way ANOVA, unless stated otherwise.

## Data availability

The mass spectrometry proteomics data have been deposited to the ProteomeXchange Consortium *via* the PRIDE partner repository with the dataset identifier PXD061808. Additional materials are available from the corresponding author upon reasonable request, including raw data used for analysis and figure generation, data about AMPK kinase assays, plasmids, and cell lines generated in this study.

## Supporting information

This article contains supporting information.

## Conflict of interest

The authors declare that they have no conflicts of interest with the contents of this article.
