# Supplementary material for: Phosphorylation of SNX17 impedes activation of Retriever-mediated sorting
Source: J Biol Chem. 2025 May 9;301(6):110222. doi: 10.1016/j.jbc.2025.110222 (PMC12182294; doi:10.1016/j.jbc.2025.110222)
Supplement: Supporting Information [file mmc1.docx]

**Phosphorylation of SNX17 impedes activation of Retriever-mediated sorting**

Jan Dominik Speidel^1^, Kaikai Yu^1^, Ralph Thomas Böttcher^1,*^

^1^Department of Molecular Medicine, Max Planck Institute of Biochemistry, Martinsried, Germany

**Supplementary Material included:**

Supplementary Figures 1-4

**
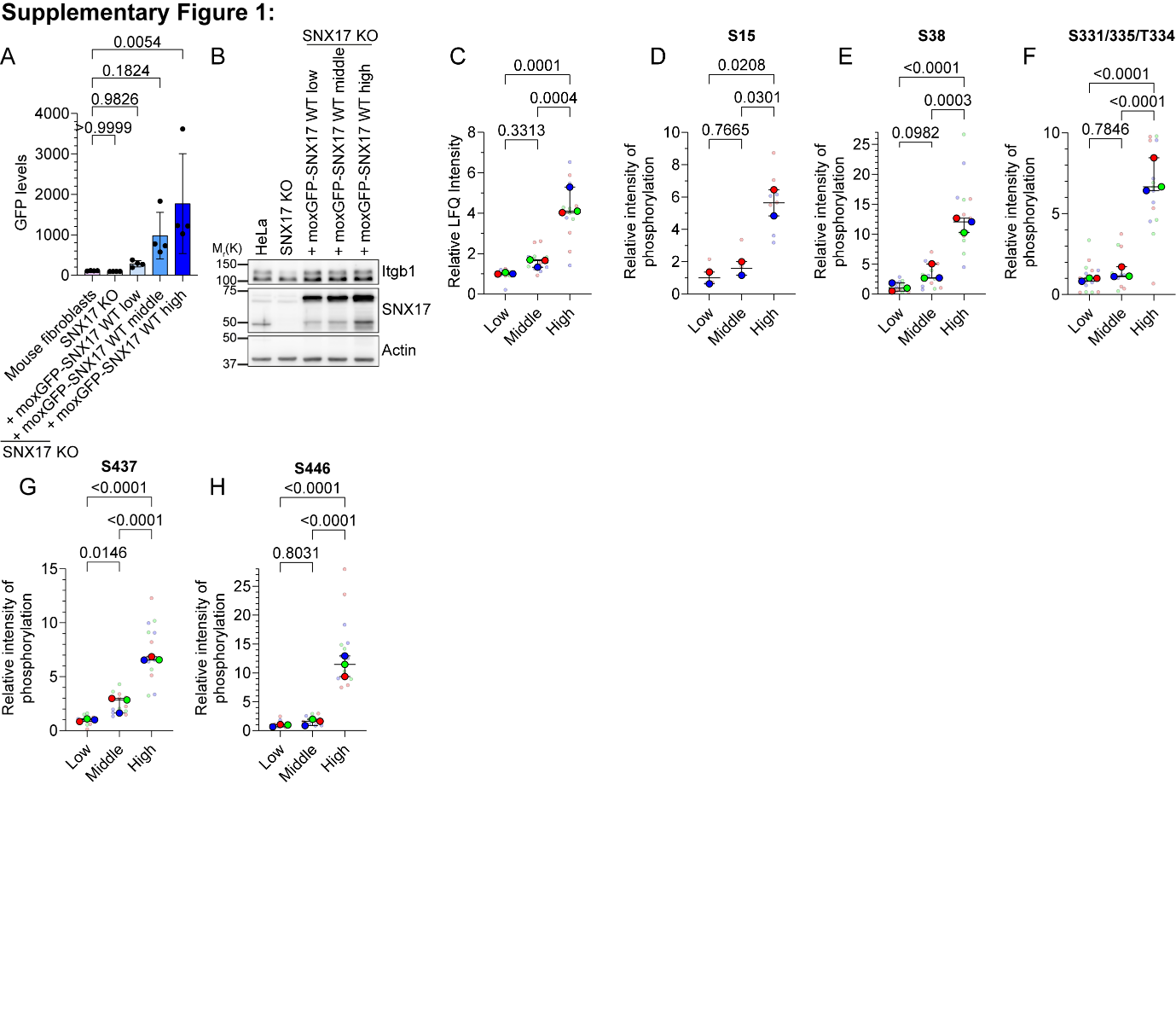
Supplementary Figure 1. SNX17 expression level affects its phosphorylation status.**

(A) Quantification of GFP levels in SNX17 KO fibroblasts re-expressing different levels of moxGFP-tagged wild-type SNX17 by flow cytometry (mean ± s.d.; n=4). Statistical analysis was carried out with one-way ANOVA + Dunnett multiple comparison test compared to surface levels of mouse fibroblasts. (B) Western blot analysis of wild-type and SNX17 KO HeLa cells, and SNX17 KO lines re-expressing different levels of moxGFP-tagged wild-type mouse SNX17. Actin served as loading control. (C-H) Relative label-free quantitation (LFQ) levels (C) and relative intensities of phosphorylated peptides of the phospho-sites S15 (D), S38 (E), S331/335/T334 (F), S437 (G), and S446 (H) of mouse SNX17 in SNX17 KO HeLa cells stably re-expressing different levels of moxGFP-SNX17 WT (median ± range; n=3 with five technical replicates each). Statistical analysis was carried out with one-way ANOVA + Tukey multiple comparison test compared to the low expression cell line.


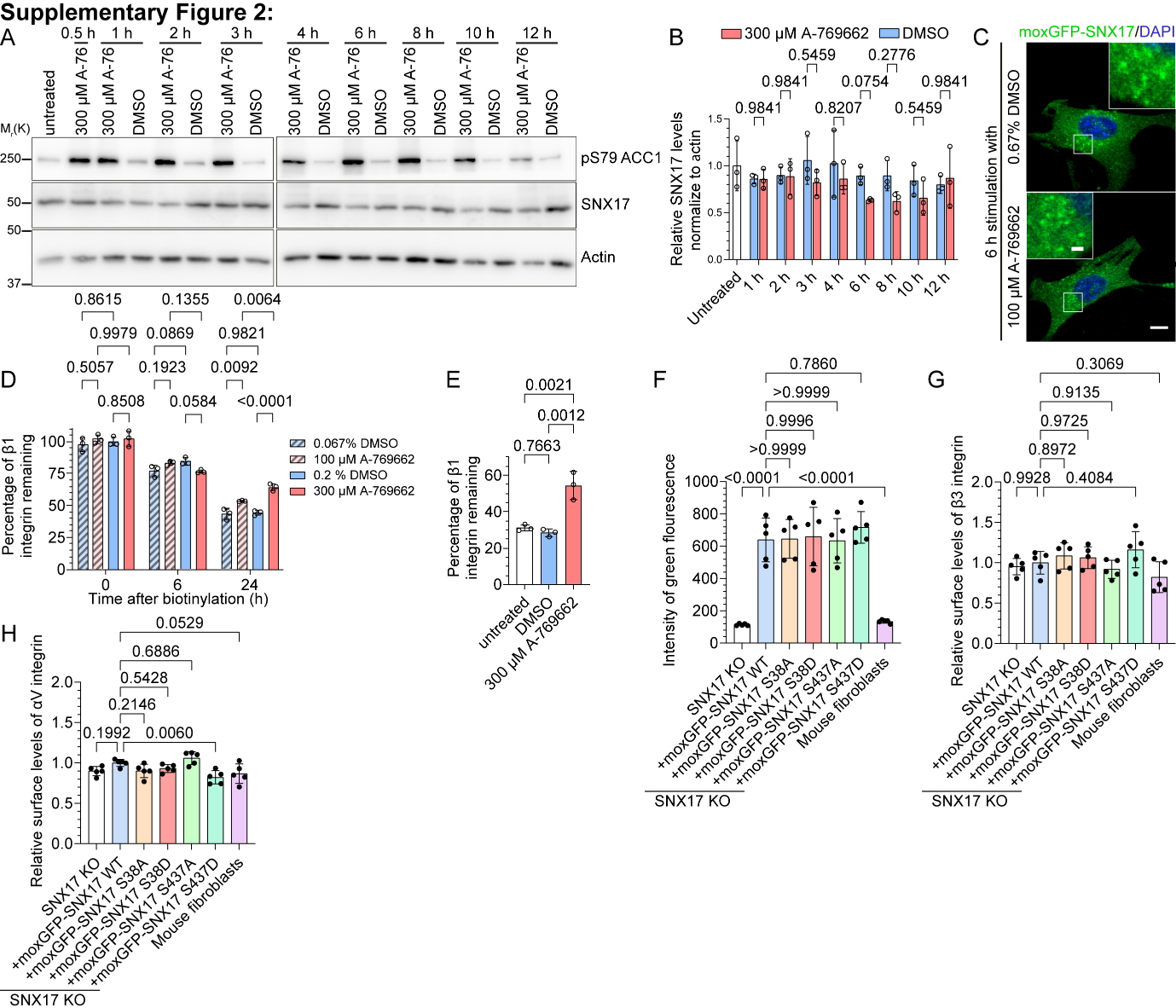


**Supplementary Figure 2. Effect of AMPK activation on SNX17 levels and integrin stability.**

(A) Western blot analysis of SNX17 levels in U2OS cells stimulated with 300 µM of AMPK activator A-769662 (A-76) or 0.2% DMSO (v/v) as vehicle control for the indicated time points. pS59 ACC1 served as marker for AMPK activation and actin served as loading control. (B) Quantification of SNX17 levels normalized to actin after stimulating U2OS cells with A-769662 or as control with 0.2% DMSO (v/v) for the indicated time points (mean ± s.d.; n=3). Statistical analysis was carried out with unpaired multiple t-tests + two-stage step-up method of Benjamini, Krieger and Yekutieli compared to each DMSO control. (C) Subcellular localization of moxGFP-tagged wild-type SNX17 6 h after stimulation with AMPK activator A-769662 or DMSO as control. DAPI was used to stain nuclei. Scale bar: 10 μm; 2 µm for magnified inserts. (D) Quantification of cell surface Itgb1 stability in fibroblasts 24 h after stimulation with the AMPK activator A-769662 or DMSO as control. Cells were pre-stimulated for 2 h, followed by biotinylation (0 h) and further stimulation in DMEM + 1% FBS for 24 h (mean ± s.d. relative to Itgb1 stability after 0 h stimulating 0.2% DMSO; n=3). Statistical analysis was carried out with two-way ANOVA + Tukey multiple comparison test. (E) Quantification of surface Itgb1 degradation in SNX17 KO fibroblasts 24 h after stimulation with AMPK activator A-769662 or DMSO as control. Cells were pre-stimulated for 2 h, followed by biotinylation (0 h) and further stimulation in DMEM + 1% FBS for 24 h (mean ± s.d; n=3). Statistical analysis was carried out with one-way ANOVA + Tukey multiple comparison test. (F-H) Quantification of GFP levels (F) or surface levels of Itgb3 (G) and ItgaV (H) on wild-type and SNX17 KO fibroblasts, and SNX17 KO fibroblasts stably re-expressing the indicated moxGFP-tagged SNX17 variants by flow cytometry (mean ± s.d.; n=5). Statistical analysis was carried out with one-way ANOVA + Dunnett multiple comparison test compared to surface levels of moxGFP-SNX17 WT expressing cell line.


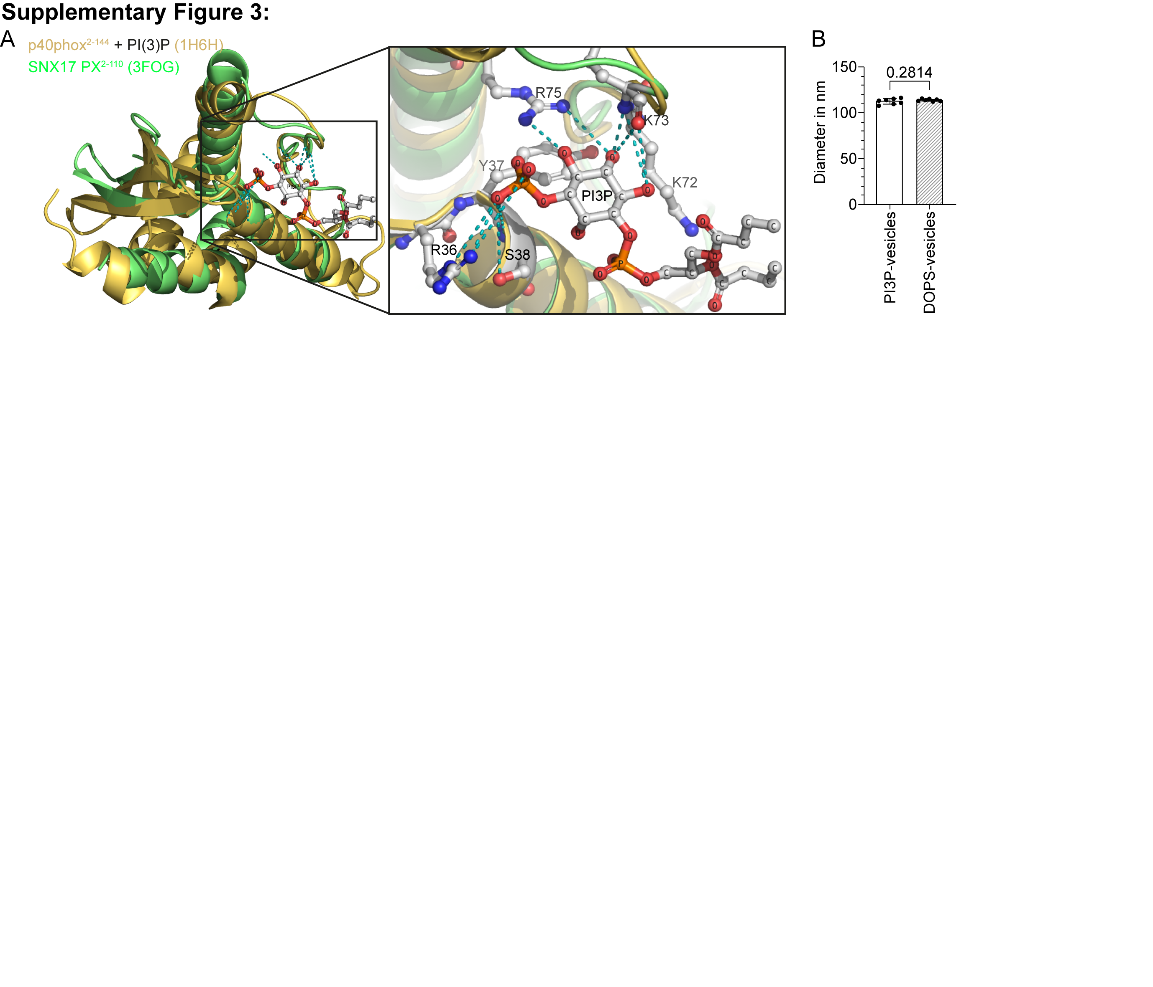


**Supplementary Figure 3. SNX17 S38D mimicking phosphorylation does not bind to PI3P.**

(A) Structure alignment of the PX domain from Neutrophil cytosol factor 4 (p40phox) bound to PI3P (PDB ID: 1H6H) and the PX domain from human SNX17 (green; PDB ID: 3FOG). Magnification shows computed hydrogen bonds between PI3P and the PX domain of SNX17. (B) Representative analysis of unilaminar vesicle diameter determined by dynamic light scattering (mean ± s.d.; technical replicates n=7). Statistical analysis was carried out with unpaired t-test (two-tailed) with 95% confidence interval.


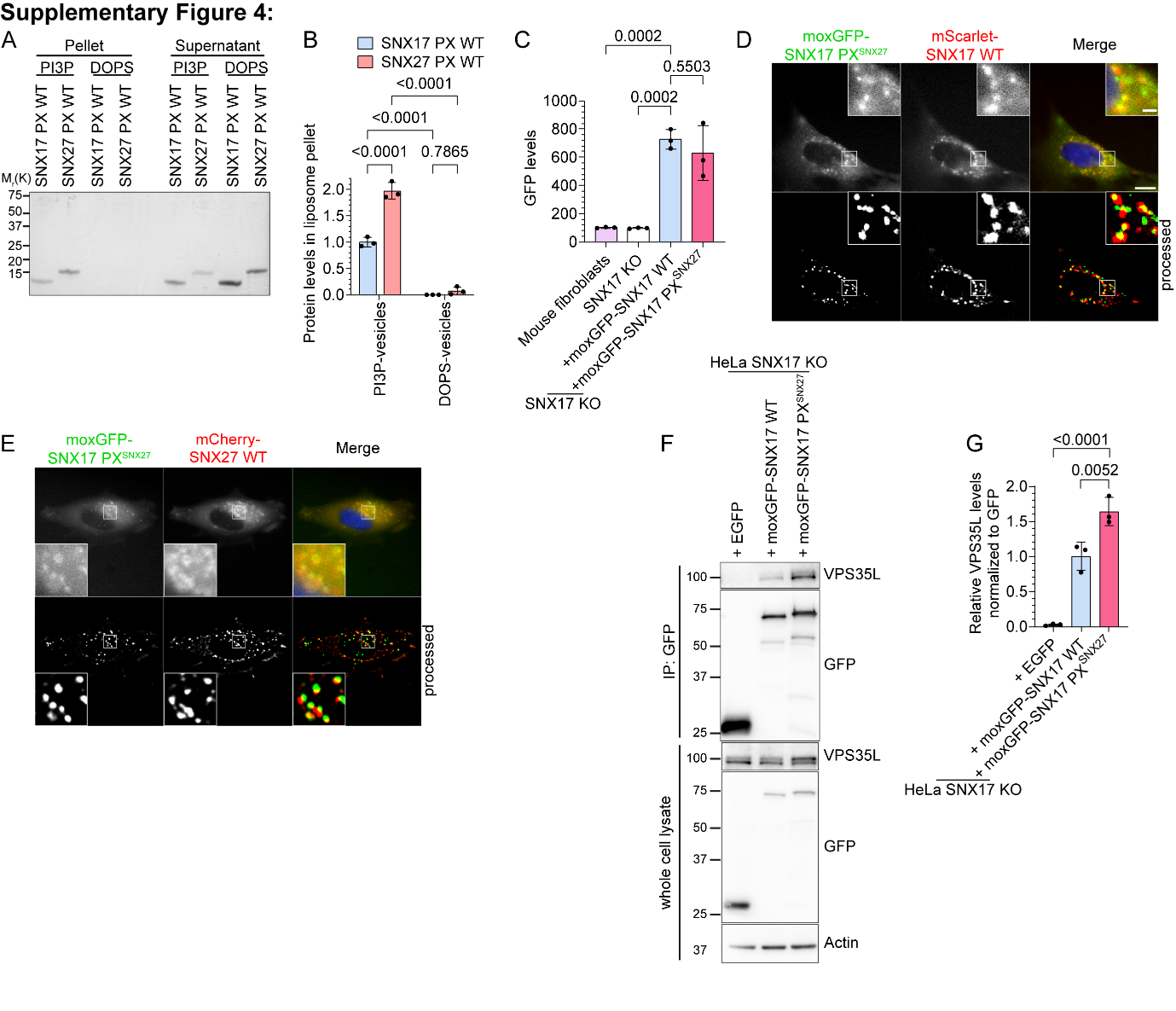


**Supplementary Figure 4. Mutations in the PX domain of SNX17 influence the binding of the cargo.**

(A) Representative gel Coomassie staining of liposome pelleting assay comparing the binding of mouse SNX17 PX WT and human SNX27 PX WT. (B) Quantification of PX domain binding to liposomes. The levels of SNX17 PX WT and SNX27 PX WT in the pellet fraction are plotted relative to SNX17 PX WT binding to PIP3-vesicles (mean ± s.d.; n=3). Statistical analysis was carried out with two-way ANOVA + Tukey multiple comparison test compared to SNX17 PX WT. (C) Analysis of moxGFP-SNX17 levels in wild-type and SNX17 KO fibroblasts, and SNX17 KO cells stably re-expressing indicated SNX17 PX variants by flow cytometry (mean ± s.d.; n=3). Statistical analysis was carried out with one-way ANOVA + Dunnett multiple comparison test compared to cells expressing moxGFP-SNX17 WT. (D,E) Represented images of moxGFP-tagged SNX17 PX^SNX27^ (green) and mScarlet-tagged SNX17 (red) (D) or mCherry-tagged SNX27 (red) (E) in SNX17 KO fibroblasts. Cell nuclei were stained using Hoechst 33342. Living cells were imaged on a wide-field fluorescence microscope. Denoised images with background subtraction are marked with “processed”. Scale bars: 10 μm; 2 µm for zoom area. (F) Western blot analysis of whole cell lysates and GFP immunoprecipitations of SNX17 KO Hela cells stably expressing EGFP or moxGFP-tagged SNX17 WT or SNX17 PX^SNX27^. Blots were probed with antibodies against GFP and VPS35L to analyse the interaction between SNX17 and VPS35L. (G) Quantification of co-immunoprecipitated VPS35L normalized to GFP signal.
